# State of the art medical devices for fluorescence-guided surgery (FGS): technical review and future developments

**DOI:** 10.1007/s00464-024-11236-5

**Published:** 2024-09-18

**Authors:** Alessandra Preziosi, Cecilia Cirelli, Dale Waterhouse, Laura Privitera, Paolo De Coppi, Stefano Giuliani

**Affiliations:** 1 Cancer Section, Developmental Biology and Cancer Programme, UCL Great Ormond Street Institute of Child Health, 30 Guilford Street, London, WC1N 1EH UK; 2 University College London, Wellcome/EPSRC Centre for Interventional and Surgical Sciences, London, UK; 3 Department of Paediatric Surgery, Fondazione IRCCS Ca’ Granda Ospedale Maggiore Policlinico Di Milano, Milan, Italy; 4 Academic Paediatrics, Imperial College Healthcare NHS Trust, London, UK; 5 Great Ormond Street Hospital for Children NHS Foundation Trust, 5th Floor Paul O’Gorman Building, Great Ormond Street, London, WC1N 3JH UK

**Keywords:** Fluorescence-guided surgery medical devices, Optical imaging, Indocyanine Green

## Abstract

**Background:**

Medical devices for fluorescence-guided surgery (FGS) are becoming available at a fast pace. The main challenge for surgeons lies in the lack of in-depth knowledge of optical imaging, different technical specifications and poor standardisation, and the selection of the best device based on clinical application.

**Methods:**

This manuscript aims to provide an up-to-date description of the commercially available fluorescence imaging platforms by comparing their mode of use, required settings, image types, compatible fluorophores, regulatory approval, and cost. We obtained this information by performing a broad literature search on PubMed and by contacting medical companies directly. The data for this review were collected up to November 2023.

**Results:**

Thirty-two devices made by 19 medical companies were identified. Ten systems are surgical microscopes, 5 can be used for both open and minimally invasive surgery (MIS), 6 can only be used for open surgery, and 10 only for MIS. One is a fluorescence system available for the Da Vinci robot. Nineteen devices can provide an overlay between fluorescence and white light image. All devices are compatible with Indocyanine Green, the most common fluorescence dye used intraoperatively. There is significant variability in the hardware and software of each device, which resulted in different sensitivity, fluorescence intensity, and image quality. All devices are CE-mark regulated, and 30 were FDA-approved.

**Conclusion:**

There is a prolific market of devices for FGS and healthcare professionals should have basic knowledge of their technical specifications to use it at best for each clinical indication. Standardisation across devices must be a priority in the field of FGS, and it will enhance external validity for future clinical trials in the field.

**Graphical abstract:**

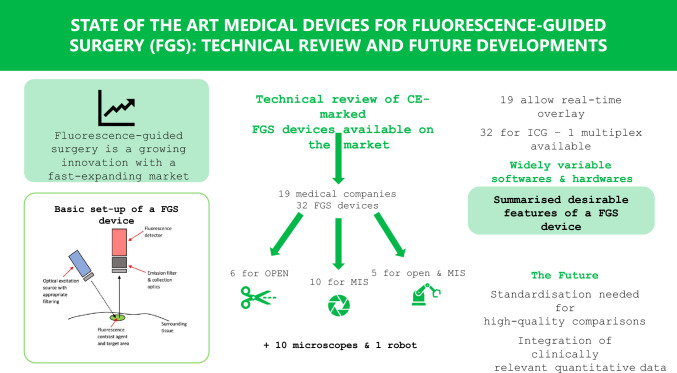

Fluorescence-guided surgery (FGS) is a real-time imaging technique that aids the visualisation of different tissues and structures during surgical intervention [1]. This new technology relies on the use of non-invasive optical imaging to guide the surgeon intra-operatively whilst avoiding radiotoxicity [2]. Fluorescence imaging is characterised by high contrast, high sensitivity, and good tissue penetration depth; the required devices to image fluorescence are portable and more affordable than alternative intra-operative imaging techniques [1]. Tissue visualisation is achieved by utilising a fluorescent contrast agent (fluorophore), which emits light when excited using a specific wavelength [3]. The majority of fluorophores currently in clinical use for FGS are imaged using the “first window” of the near-infrared light (NIR-I), which covers wavelengths from 650 to 900 nm and it is associated with low scattering and reduced tissue autofluorescence [4, 5]. The most used injectable fluorophore for FGS is Indocyanine Green (ICG; peak excitation: ≈ 780 nm and peak emission: ≈ 830 nm), a water-soluble tricarbocyanine dye that readily binds to plasma proteins, undergoes hepatic metabolism and biliary elimination [3].

The basic components of an FGS medical device are illustrated in Fig. 1. A light source with appropriate filters excites the fluorophore; the emitted signal from the probe then passes through emission filters and is collected by optics; thereafter, this is focused on a detector and ultimately transferred to a computer for visualisation [1]. Excitation sources used for fluorescence imaging systems mainly consist of broadband lights such as xenon lamps, laser diodes (LD), and light-emitting diodes (LED) [5]. Typically, LDs are more expensive but exhibit strong wavelength selectivity and a narrow bandwidth; they have a higher intensity than LED [4]. LEDs are the most commonly used excitation source thanks to their lower price, long lifespan, and low electricity consumption [4]. Operating theatre illumination may significantly impact image acquisition, and special care is required to reduce noise signal caused by environmental light. The confined spectrum in the visible light band of LEDs makes these the ideal surgical room lighting, causing less interference with the fluorescence signal. Room light sources such as tungsten, halogen bulbs, and sunlight, should be turned off during the fluorescence imaging [6]. Emission filters and collection optics are another essential component of an FGS system. Emission filters with larger bands enhance the collection of fluorescence signal; however, they increase autofluorescence and reduce the image quality due to unwanted recorded signals [1]. The optical path from emitting fluorescence signal to the image detector determines the working distance, the field of view, and the image contrast [4]. To detect the image, cost-efficient charge-coupled device (CCD)-based cameras are most commonly used for fluorescence imaging despite their low quantum efficiency in the NIR wavelength range and slow digitisation rate (< 30 Hz) [6]. Yet, low-light conditions lead to low signal and exacerbate the CCDs’ low efficiency and slow read-out time. Electron multiplied CCDs and intensified CCDs can overcome this challenge by amplifying the signal in an electron-multiplication register [7].

**Fig. 1:**
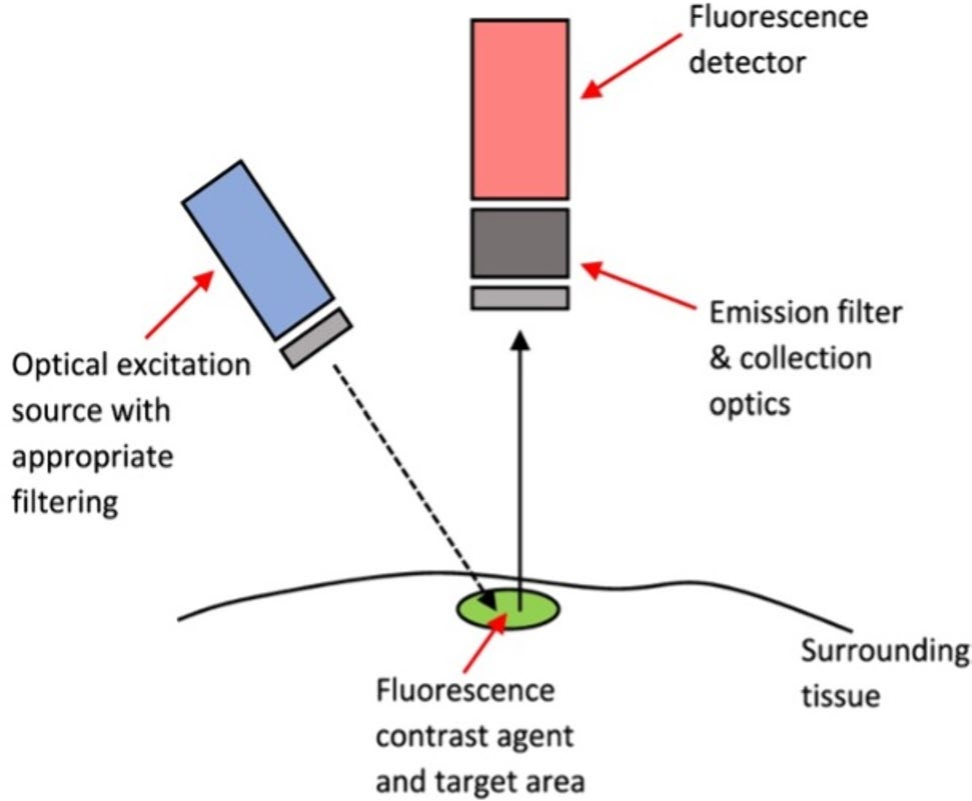
Schematic representation of the set-up of an FGS system (Image from Ref. [1], published under CC BY 4.0 license)

Different medical devices can provide different imaging visualisation modalities, including a black-and-white (monochromatic) fluorescence image, a coloured fluorescence heatmap, and an overlay of white light and fluorescence images (Fig. 2). The last visualisation modality is a particularly desirable feature as it enables a more detailed and comprehensive delineation of the surgical field; this aims to simultaneously augment the surgeon’s vision with fluorescence, without obscuring native information [8, 9].

**Fig. 2:**
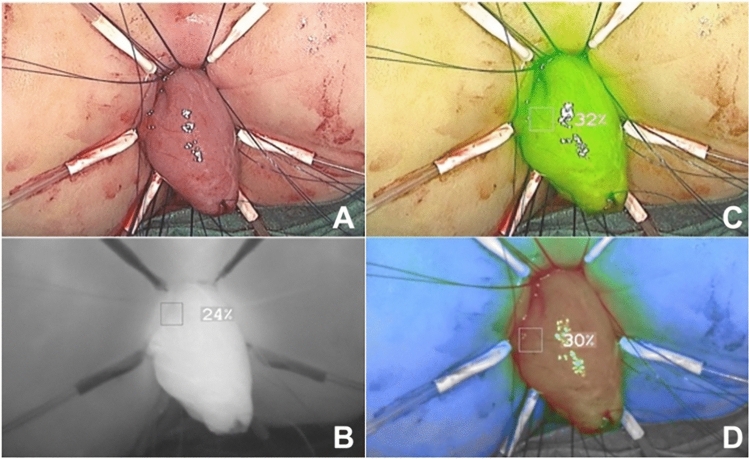
FGS image types. The different image types that can be provided by an FGS system are displayed here: white light (**A**), monochromatic fluorescence image (**B**), a green fluorescence image overlay (**C**), and a fluorescence heatmap (**D**). The images were captured with the SPY-PHI® device by Stryker during a rectal pull-through to treat total colonic aganglionosis (written parental consent was granted for the inclusion of these images in the manuscript)

Imaging platforms can be designed with different levels of complexities, which include the use of beam splitters and a combination of multiple cameras or multispectral cameras, which separate visible light from NIR wavelengths using prisms and multiple CCDs [6]. During FGS, it is important to stabilise the device at 15–20 cm away from the target to achieve best fluorescence intensity and avoid false negative images.

Multispectral cameras allow the simultaneous or near-simultaneous (through channel-switching) visualisation of multiple fluorophores across several wavelengths. The combination of different fluorophores is an appealing future development in the field, as it could be used to visualise simultaneously and with different colours several anatomical and/or pathological structures (Fig. 3), or to separate distinct molecular signatures within a single lesion [10].

**Fig. 3:**
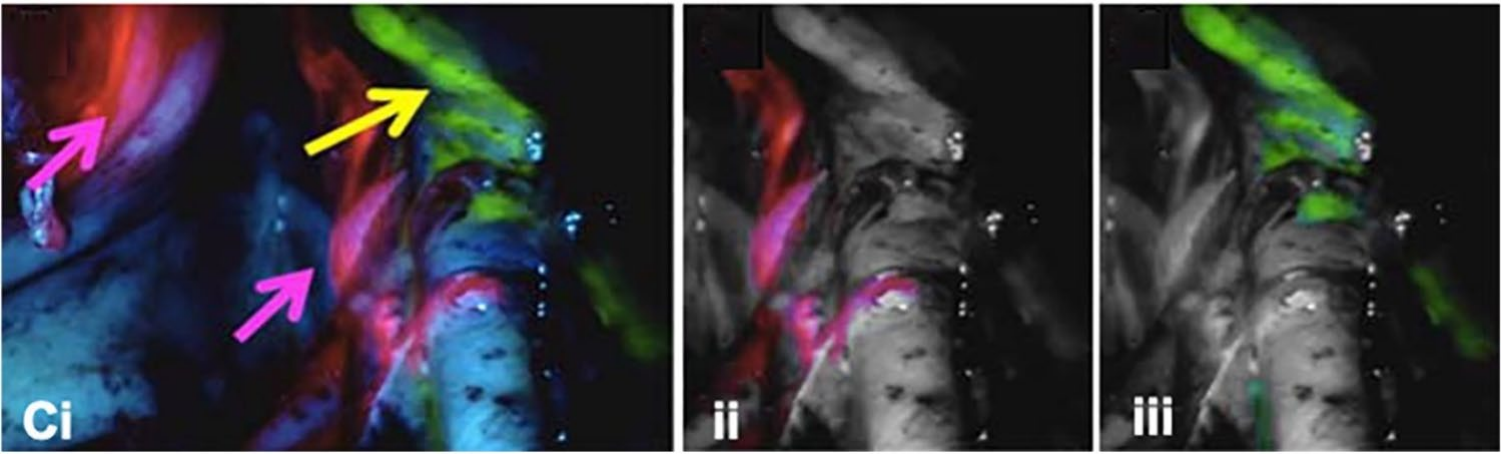
Multi-wavelength image of lymphatic ducts. Multispectral image simultaneously displaying ICG-stained nodes (pink arrows) and fluorescein-stained lymph duct (yellow arrow) (Ci). Images digitally separating the 2 signals into ICG (ii) and fluorescein (iii) (Image adapted with permission from research originally published in J Nucl Med, P. Meershoek et al. © SNMMI [10])

The popularity of ICG-based FGS has grown over the past decades across a variety of surgical fields; applications span from the assessment of tissue perfusion in plastic and colorectal surgery, to the identification of anatomical structures in hepatobiliary and genitourinary surgery, and lymph node targeting and imaging of cancer tissue [9–11]. A recent consensus by the European Association for Endoscopic Surgery (EAES), on review of the literature, strongly recommended FGS use to aid anatomical identification and reduce complications in laparoscopic cholecystectomy, colorectal perfusion assessment, and sentinel lymph node search in gynaecological malignancies. In all these fields, the use of ICG is perceived as improving the precision of the surgical technique, the identification of the blood vessels and the lymph node detection rate, allowing for better image quality compared with standard white light [12].

The growing popularity of FGS is driving established and new medical device companies to compete in the production of high-tech devices with more features and set-up options. Despite recent advancements, most surgeons lack crucial knowledge of optical imaging or have not yet used FGS. The aim of this review is to provide an up-to-date overview of the surgical fluorescence medical devices available on the market. Their widely variable technical characteristics will be compared to support the surgeons to choose and use appropriately the most suitable platform for their clinical needs.

## Methods

A broad search was conducted in the PubMed database, using a combination of the following keywords: “fluorescence guided surgery”, “ICG device”, “NIR camera”, “fluorescence system”, “imaging device”, and “fluorescence camera”. Moreover, medical companies were directly contacted for more details on their fluorescence imaging device. Data was collected for medical devices available on the market in November 2023. Surgical devices with insufficient available information non-CE-marked were excluded from this review. Some manufacturers did not respond to our requests for information on their device; in these cases, we have only shared information obtained from published literature or online. The devices were compared according to the mode of use, basic hardware components, settings required, image modalities, compatible fluorophores, regulatory approval, clinical indications, and cost. The authors have no financial interests in the devices described in this review.

## Results

### Mode of use of devices

**Fig. 4:**
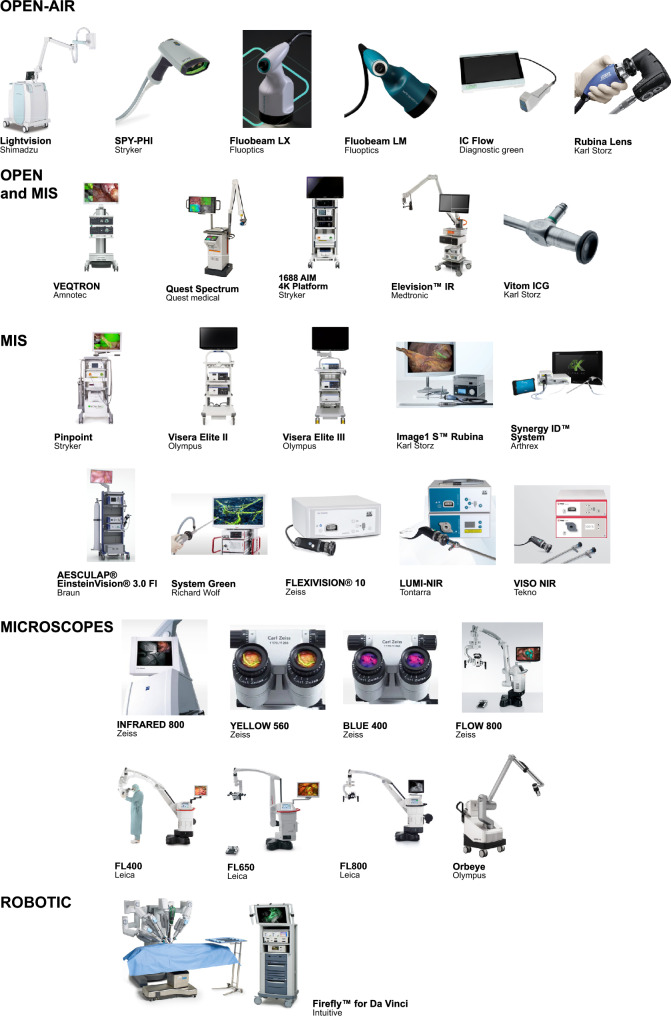
The fluorescence-guided surgery devices available on the market up to November 2023. The upper panel shows the devices available for open, minimally invasive, and robotic surgery, whilst the lower panel shows the surgical microscopes available on the market: GLOW400 and GLOW800 are not displayed as they are digital accessory

This review’s search identified 32 FGS devices with CE-marking for use in the clinical environment. Each device was first assigned to a specific “device category” based on its clinical use. The 5 categories were as follows: open surgery, minimally invasive surgery (MIS), both open and MIS surgery, robotic surgery, and surgical microscopes (Fig. 4).

Six devices, FLUOBEAM LM® (*Fluoptic*s®), FLUOBEAM LX® (*Fluoptic*s®), SPY-PHI® (*Stryker)*, LIGHTIVISION (*Shimadzu*), IC Flow™ (*Diagnostic Green*) and Rubina® Lens (*Karl Storz*) can be used for open surgery; their clinical applications include plastic, lymphatic, thyroid, and liver surgery [13, 14]. These systems rely on hand-held devices, compact and easy to grip during surgery. The excitation sources are designed to work within ICG wavelength. All these devices need the room lights to be off or very low to avoid interference with the fluorescence signal.

Five devices provide both open and MIS modes (Quest Spectrum®, Vitom® ICG, Elevision™ IR Platform, 1688 AIM 4K Platform, and VEQTRON®), and 10 are available only for MIS (Pinpoint®, Image1 S™ Rubina®, Visera Elite II, Visera Elite III, AESCULAP® EinsteinVision® 3.0 FI, System green, Flexivision®, Lumi-NIR, Viso NIR, and Synergy^ID^™). Firefly™ for Da Vinci (*Novadaq/Intuitive*) is the only system integrating fluorescence imaging in robotic surgery. The remaining 10 fluorescence systems consist of surgical microscopes, provided by *Leica, Zeiss,* and *Olympus*; they are mainly used in neurosurgery, although some are also utilised in plastic, cardiovascular surgery, and urology.

### Basic hardware and setting

An imaging system should operate under normal room lights to be easily adaptable to a surgical suite environment. However, the operating lights can often interfere with the fluorescence signal, and it is preferable to turn them off whilst assessing fluorescence. This represents a current challenge, especially in open surgical procedures. To overcome this issue, some devices can perform background correction by pulsing the LED or LD excitation light source in a synchronised manner to a gated or shuttered detector system, such as CMOS (Complementary-Metal–Oxide–Semiconductor) or ICCD camera [15]. Similar background mitigation can be achieved using frequency modulation and lock-in detection [16].

Amongst the devices available for open and MIS surgery, only 4 devices can be utilised with the operating lights on: LIGHTVISION (*Shimadzu*), 1688 AIM 4K Platform (*Stryker*), Elevision™ IR Platform (*Medtronic*) and Quest Spectrum® (*Quest medical*). The Elevision™ has two different illumination sources merging on the operative field through a cable: a xenon pure white light source and 805/785 nm laser excitation. The 805 nm laser exclusively excites ICG, leading to a more sensitive fluorescent signal compared to the systems using filters as the excitation sources.

### Image types and fluorophores

Providing an overlay between the fluorescent and white light images is one of the most desirable features of fluorescence imaging platforms. Based on our search, there are 19 systems able to provide the image overlay in real-time. Amongst the open-air devices, SPY-PHI® (*Stryker*), LIGHTVISION (*Shimadzu*) and Rubina® Lens (*Karl Storz*) have this feature. SPY-PHI® provide various visualisation modalities: white light image, monochromatic fluorescence mode, green fluorescence overlay mode, and colour-segmented fluorescence mode. LIGHTVISION can provide 3-windows display where white light image, NIR-image and visible light + NIR images can be seen simultaneously. Rubina® Lens offers 4K image quality as well as an intensity map of the NIR/ICG signal.

Amongst the open and MIS systems, Quest Spectrum®, Elevision™ IR Platform, 1688 AIM 4K Platform and VEQTRON® can also provide the overlay image. Image1 S™ Rubina®, Pinpoint®, Visera Elite II and III, Synergy^ID^™, AESCULAP® EinsteinVision® 3.0 FI, SystemGreen amongst the MIS devices, and GLOW800, FL560, INFRARED 800, YELLOW 560, and FLOW 800 amongst surgical microscopes have this feature, too.

The information captured with the Quest Spectrum® can be visualised within various fluorescence formats. The screen can be set up on a “full-screen” mode displaying one selected image view, or an “expanded mode” showing 4 images simultaneously—the standard whole-coloured white light image, the monochromatic image, the green fluorescence overlay, and the merged white light and fluorescence images visible with different gradient overlay depending on signal intensity. The expanded mode allows the surgeon to quickly access all the different image outputs and to select any of them to be viewed in full screen mode. The Elevision IR™ provides similar features; this imaging platform comes with an easy-to-handle camera for open surgery and a supporting arm, allowing surgeons to move it around the field. The camera has been designed with integrated buttons that allow direct control of the main monitor. The surgeon can select between 3 image modalities: the monochromatic image, the green-overlay image, and the fluorescence-only image. There is also a fourth modality called “relative30”; this allows the visualisation of the tissues that express less than 30% of the maximum fluorescence detected and are therefore more likely to be poorly perfused. The Elevision IR™ has been used in paediatric surgery [17], plastic surgery [18], urology [19], and thyroid surgery [20]. The SPY-PHI® and Elevision™ IR Platform are two devices amongst the ones suitable for open surgery capable of quantifying fluorescence intensity parameters; these platforms can show in real-time the percentage fluorescence of a selected region when compared to a set reference maximum fluorescence [18].

The *Storz* recommended set for ICG-enhanced fluorescence-guided laparoscopy is the Image1 S™ Rubina® technology, which includes a 4K camera and is based on the IMAGE S™ camera platform. This system provides surgeons with a high-quality 4K video and 3D visualisation, and 3 different NIR/ICG available modes (overlay, intensity map, and monochromatic). The overlay mode offers the possibility to view the images either in blue or green: the green colour provides a detailed delineation against the surrounding tissue, whilst the blue colour allows a more balanced fluorescence image. Image1 S™ Rubina® is also user-friendly; a foot-pedal control or camera head buttons allow the surgeons to easily switch between standard white light mode and NIR/ICG mode. The system offers multidisciplinary applications, including paediatric surgery [21], colorectal surgery, surgical oncology, and lymph node detection [22]. The Image1 S™ system by *Storz* offers expanded compatibility for applications in endoscopy and open surgery. Indeed, VITOM® ICG (*Storz*) can be used with components of the ICG system and be integrated into the surgeon’s operating field using a holding arm to adapt its use to open surgery. The Synergy^ID^™ device produced by *Arthrex®* features a bifurcated LED and laser light source and an ultra-high definition 4K camera, thanks to the Synergy^UHD4^™ camera system and four imaging sensors. This device can display 3 ICG-fluorescent image types—a green fluorescence overlay, a monochromatic image, and a monochromatic and NIR merged image. The camera head button or tablet device can be used to effortlessly switch between the image types. This device has been used in biliary anatomy delineation, bowel perfusion assessment, ureter identification, and cardiothoracic procedures.

Most of the systems described are designed to work within ICG wavelength (780–830 nm). Only Quest Spectrum® is multiplex available and is being developed to also work with methylene blue by incorporating two imaging channels; this demonstrates more versatility than the other devices. Quest Spectrum® has already been used for perfusion assessment in breast reconstruction and colorectal surgeries [20, 21]; oncological applications are yet to be explored, although the multiplex availability makes this device valuable in the field of research to trial emergent cancer probes.

ICG-fluorescence imaging can support compatible devices to aid surgical interventions in the fields of neurosurgery, ENT, and plastics; in fact, surgical microscopes can facilitate the resection of malignant tissue, by aiding maximum removal of abnormal cells with minimal impact on the surrounding tissue, and the assessment of blood flow. FL800 by Leica enables the surgeon to observe blood flow through the microscope eyepieces or on the video monitor in real-time after ICG injection; overlay is not available. GLOW800 and GLOW400 (*Leica*) can be considered an upgrade, providing a 3D digital overlay of white light and NIR image. Certainly, this contributes to the higher cost of GLOW when compared to FL800. FL400 (*Leica*) and FL560 (*Leica*) differ from other devices, as they are just filters that can be integrated into the microscope. They work within wavelengths of 380–430 nm and 460–500 nm, respectively. Therefore, they can be used to visualise 5-aminolevulinic acid (5-ALA) to differentiate the tumour’s bulk and its margins. Exogenous 5-ALA is administered orally and is taken up by malignant glioma cells; it is then metabolised into the fluorescent protoporphyrin IX (PpIX). Elevated PpIX level within malignant brain tumour cells allows violet-red fluorescence visualisation of tumour tissue after excitation with the 405 nm wavelength light [23]. FL400 allows direct viewing through the binoculars, as no cameras are involved in observing light.

*Zeiss’* INFRARED 800 consists of optics for the excitation in the 700–800 nm wavelength range and a fully integrated camera into the surgical microscope. It is easily activated with a button, ultimately delivering greyscale images. INFRARED 800 is currently used for intra-operative visual assessment of blood flow and vessel patency during arteriovenous malformation, bypass, and aneurysm resection. FLOW 800 (*Zeiss*) is a software for analytical colour visualisation and evaluation of fluorescence signals obtained by the microscope; a coloured map identifies the direction and the intensity of blood flow over time [24]. BLUE 400 (*Zeiss*) and YELLOW 560 (*Zeiss*) can be integrated into the surgical microscope. The first filter works in the 400–410 nm wavelength range for excitation and 620–710 nm wavelength range for emission. The second filter is designated for excitation in the wavelength range from 460 to 500 nm and for observation in the wavelength range from 540 to 690 nm; for this reason, this kit has a higher specificity and requires a smaller dose of dye. According to the literature, whilst using the BLUE 400 system, the area surrounding the fluorescent tissue is dark; therefore, switching the microscope to normal illumination is necessary to control tumour vessel supply and haemostasis. On the other hand, the YELLOW 560 allows an improved delineation of the normal area surrounding fluorescent tissue; this allows the completion of the operation without switching to normal white light illumination [25].

### Regulatory approval and costs

Analysing the cost of fluorescence imaging platforms, the MIS-only and open surgery only devices are the most affordable, whilst the microscopes are the most expensive. It is important to note that the prices stated are only indicative and might not reflect current pricing; prices may also vary depending on geographical location. All prices have been converted to an approximate value in US dollars to aid comparison between devices. The indicative price of an open-surgery-only FGS device averages around 84,000 $; the price of the devices available for both open and MIS ranges between 79,000 $ and 150,000 $. The price of FGS surgical microscopes is approximately 200,000 $. All the devices are CE-marked, and 30 are FDA-regulated. All the described features are summarised in Table 1.

**Table 1:** Summary of the features of FDA-approved NIR devices available on the market

Device	Company	Image overlay	Room lights	Other features	Response
**OPEN-AIR**					
FLUOBEAM LM®	*Fluoptics*	No	Low	Easily handheld	Yes
SPY PHI®	*Stryker*	Yes	Low	Easily handheld	Yes
FLUOBEAM LX®	*Fluoptics/inomed*	No	Low	Torch-like, easily handheld	Yes
LIGHTVISION	*Shimadzu*	Yes	On	–	No
IC Flow™	*Diagnostic Green*	No	Low	Easily handheld	Yes
Rubina® Lens	*Karl Storz*	Yes	Low	Easily hand held	Yes
**MIS**					
Pinpoint®	*Stryker*	Yes	Low	–	Yes
Visera Elite II	*Olympus*	Partial	Low	Magenta glow overlay	Yes
Visera Elite III	*Olympus*	Yes	–	3D, software-based	Yes
Image1 S™ Rubina®	*Karl Storz*	Yes	Low	4K and 3D	Yes
Synergy ID™	*Arthrex*	Yes	Low	4K and 3D	Yes
AESCULAP® EinsteinVision® 3.0 FI	*Braun*	Yes	Low	3D	Yes
SystemGreen	*Richard Wolf*	Yes	–	4K, 2 image modes	No
FLEXIVISION	*Scholly*		–	–	No
Lumi-NIR	*Tontarra*	No	–	–	No
VISO NIR	*Tekno*	No	–	4K	No
**OPEN and MIS**					
1688 AIM 4K Platform	*Stryker*	Yes	On	3 images modes	Yes
Quest Spectrum®	*Quest Medical*	Yes	On	Multiplex/4 image modes	Yes
Vitom® ICG	*Karl Storz*	No	Low	Easily handheld	Yes
Elevision™ IR Platform	*Medtronic*	Yes	On	3 image modes	Yes
VEQTRON®	*Amnotec*	Yes	Low	3D, adjustable sensitivity, 3 image modes	Yes
**ROBOTIC**					
Firefly™ for Da Vinci	*Novadaq/Intuitive*	No	Low	–	No
**MICROSCOPES**					
FL800	*Leica*	No	On	No overlay	Yes
GLOW400	*Leica*	Yes		Digital accessory	Yes
GLOW800	*Leica*	Yes	On	Digital overlay	Yes
FL560	*Leica*	Yes	On	–	Yes
FL400	*Leica*	No	On	–	Yes
Infrared 800	*Zeiss*	Yes	On	Compact handheld design	Yes
Yellow 560	*Zeiss*	Yes	On	–	Yes
Blue 400	*Zeiss*	No	On	–	Yes
Flow 800	*Zeiss*	Yes	On	Software for surgical microscope	Yes
ORBEYE	*Olympus*	No	Low/on	4K, 3D–ICG & 5-ALA	No

## Discussion

The exponential growth in the field of FGS is exemplified by major medical companies releasing flagship fluorescence imaging devices at unprecedented rates. From suitable fluorophore use and hardware settings, to compatibility with theatre lights and image types provided, these products boast ever-more impressive features to enhance intra-operative visualisation. This review compared the fluorescence imaging devices available on the market, to introduce surgeons to the features of a FGS system and elucidate which devices might be best suited for their case mix.

Real-time fluorescence overlay on white light images is likely to be the most valued requirement, as this allows efficient visualisation of the structure targeted by the fluorophore. More than half of the devices analysed in this review can provide the overlay image output, but only three were suitable for open surgery (SPY-PHI®, LIGHTVISION, Rubina® Lens). This feature was more widely available in MIS-only devices, with seven out of ten allowing a degree of fluorescence overlay; three of these also sported 4K images and four facilitated 3D visualisation. Similarly, producing a monochromatic fluorescent image is incredibly useful, as it allows detailed intra-operative visualisation of vessels and better contrast to visualise margins and small fluorescence areas. Simultaneous multi-fluorophore imaging capability is another pivotal feature; this can be critical in visualising dyes with different wavelengths targeting cancer cells during oncological resections [26]. Only one device with this capability was found in this review, with a price tag on the higher end of the range identified.

Indeed, price is another essential element influencing the selection of an imaging platform; the cheapest device available is approximately 31,600 $, whilst the most expensive microscope costs more than seven times as much. We observed that four devices, out of the six that could be used in open surgery, featured a hand-held design, a valuable detail when considering ergonomics and portability. The ease of switching between image modalities was often quoted by manufacturers, especially for devices suitable for MIS; the use of a pedal or buttons was most often employed. It is important to note that whilst we have suggested a set of desirable features, each system comes with positive and negative aspects and there is no single “best” system in the market. Each surgeon should carefully assess the devices’ features, to select the most appropriate one for their practice.

The identified devices were highly variable in their features and built-in software for image visualisation; this significantly impairs comparability with an evidence-based approach. In fact, evaluating the devices’ sensitivity and specificity to detect fluorescence with different built-in software manipulating and altering the image quality is a significant challenge. This is further complicated by the variability of ICG dosing across clinical indications and patient characteristics, ranging from weight-based to fixed dose and BMI-based [27, 28]. Moreover, the variable excitation sources and emission filters chosen by different devices further challenge direct comparison in terms of peak fluorescence intensity [29].

Some FGS systems feature real-time visualisation of fluorescence parameters, such as percentage fluorescence intensity; however, the different software used to calculate these quantitative values impedes comparability between devices and their sensitivity. In the future, standardisation in the market might allow direct and valid comparison, as gold-standard features of software might be more uniformly rolled out across devices. Only then, a single “best” device might be identified for a specific clinical application or fluorophore use. However, we have described above favourable features a potential buyer might value when considering acquiring an FGS device. For the minimally invasive surgeon, ergonomics, ease of switching between image modalities, availability of real-time fluorescence overlay, and cost might be essential considerations. Certainly, the specific clinical context would also guide further decision-making, especially in the mode of use the considered device enables.

Several studies have explored the usefulness of quantitative parameters of ICG-fluorescence [30]. Most devices currently only allow a subjective analysis of the fluorescent image, relying on the individual surgeon’s interpretation of the enhanced image; yet this might have limited inter-observer agreement [31]. New evidence emphasises the value of specific features of fluorescence-time curves (FTC), such as the time to maximum fluorescence and the slope (the rate of fluorescence intensity rise to maximum levels). Promising correlations with clinical outcomes have peaked the surgeons’ and researchers’ interest in the real-time availability and validation of such parameters [32]. Yet, limited quantitative information in real-time is provided by few devices, only one of which is suitable for minimally invasive procedures; operative videos can only be retrospectively analysed with specific software to collect FTC parameters. When the clinical utility of quantitative parameters will be further emphasised by ongoing research, built-in software in FGS devices will need to provide valuable data points, to objectively aid decision-making during surgery, rather than only allowing a subjective or qualitative assessment of the fluorescent images.

It is important to acknowledge the possible limitations of this review. Due to the nature of the subject analysed, only a limited amount of information could be obtained from peer-reviewed sources after a formal search on PubMed. As noted in the methods section, a large amount of material incorporated in this review was obtained with dedicated meetings with medical companies, as disclosed in Table 1; hence, the information provided to us might have been skewed by the manufacturers. Nevertheless, the factual specifications of the devices are expected to be accurate, along with their approximate price and regulatory approval at the time of the review being conducted. In the event of not receiving a response from medical companies, we included only information available on PubMed or on publicly available product leaflets.

## Conclusion

We summarised the multitude of characteristics of the CE-approved NIR devices available on the market, describing the desirable features and suggesting criteria for selecting the most suitable imaging system for each surgeon’s need. The high degree of variability between the different FGS systems limits a direct comparison, and future research should focus on addressing this aspect. In this regard, upcoming fluorescence imaging software might benefit from real-time visualisation of quantitative parameters, to aid an objective assessment of fluorescence.

## Abbreviations

CCD: Charge-coupled devices
CE: Conformité Européene
CMOS: Complementary metal oxide semiconductor
FGS: Fluorescence-guided surgery
ICG: Indocyanine Green
LED: Light-emitting diodes
LD: Laser diodes
MIS: Minimally invasive surgery
NIR: Near-infrared
SBR: Signal-to-background ratio
